# A Prospective Multicenter Observational Study Assessing Incidence and Risk Factors for Acute Transfusion Reactions in Cats

**DOI:** 10.1111/jvim.70246

**Published:** 2025-09-29

**Authors:** Georgina B. F. Hall, Rachael Birkbeck, Benjamin M. Brainard, Fernanda Camacho, Elizabeth B. Davidow, Dana N. LeVine, Andrew Mackin, Taylor Moss, Katherine J. Nash, Giacomo Stanzani, Daria Starybrat, David Q. Stoye, Carolyn Tai, John Thomason, Julie M. Walker, K. Jane Wardrop, Helen Wilson, Virginie A. Wurlod, Karen Humm

**Affiliations:** ^1^ Clinical Science and Services, The Royal Veterinary College London UK; ^2^ The Ralph Marlow UK; ^3^ Department of Small Animal Medicine and Surgery, College of Veterinary Medicine University of Georgia Athens Georgia USA; ^4^ Willows Veterinary Centre and Referral Service Solihull UK; ^5^ Timberline Veterinary Emergency and Specialty Washington USA; ^6^ Department of Clinical Sciences, College of Veterinary Medicine Auburn University Auburn Alabama USA; ^7^ College of Veterinary Medicine, Mississippi State University Starkville Mississippi USA; ^8^ School of Veterinary Science University of Queensland Gatton Queensland Australia; ^9^ Dick White Referrals UK; ^10^ The Royal (Dick) School of Veterinary Studies University of Edinburgh Edinburgh UK; ^11^ Oxford, School of Public Health Oxford UK; ^12^ Cummings School of Veterinary Medicine Tufts University North Grafton Massachusetts USA; ^13^ Department of Medical Sciences, School of Veterinary Medicine University of Wisconsin‐Madison Madison Wisconsin USA; ^14^ College of Veterinary Medicine Washington State University Pullman Washington USA; ^15^ Langford Vets University of Bristol Bristol UK; ^16^ Louisiana State University School of Veterinary Medicine Baton Rouge Louisiana USA

**Keywords:** AHTR, feline, FNHTR, storage lesion, TACO, TRALI

## Abstract

**Background:**

The reported incidence of blood product transfusion reaction (TR) in cats varies greatly.

**Objective:**

Evaluate the incidence and practices surrounding acute TRs in cats receiving feline blood products, using Transfusion Reaction Small Animal Consensus Statement (TRACS) definitions.

**Animals:**

Cats (*n* = 444) that received feline blood products (*n* = 608) between March 2022 and January 2024.

**Methods:**

Prospective, multicenter observational study at referral hospitals (*n* = 14) in the United States, United Kingdom, and Australia documenting the incidence of acute TRs in cats receiving allogenic blood transfusions.

**Results:**

Acute TR incidence was 7.8% (95% confidence interval [CI], 5.6–10.6) for red blood cell (RBC)‐containing products and 1.1% (95% CI, 0.0–5.9) for plasma products. Febrile nonhemolytic transfusion reactions (FNHTRs) were the most common in RBC‐containing products (incidence, 5.7%; 95% CI, 3.8–8.1). Age of RBC unit (adjusted odds ratio [aOR], 1.04 per day; 95% CI, 1.00–1.07) and administering a RBC unit with an infusion pump rather than a syringe driver (aOR, 4.7; 95% CI, 1.43–15.54) were associated with an increased odds of FNHTR. Of transfusions discontinued (*n* = 28) because of a potential TR, 46.4% did not fulfill TRACS criteria for any TR. Death occurred within 24 h of a transfusion event in 18.3% but was not associated with the development of a TR (odds ratio [OR], 0.76; 95% CI, 0.29–1.99).

**Conclusions and Clinical Importance:**

Transfusion reaction incidence in cats, as defined using TRACS guidelines, is reported. Febrile nonhemolytic TRs were the most common and were associated with increasing RBC unit age and administering RBC via an infusion pump.

**Abbreviations**
AHTR: acute hemolytic transfusion reaction
aOR: adjusted odds ratio
AVHTM: Association of Veterinary Hematology and Transfusion Medicine
FFP: fresh‐frozen plasma
FNHTR: febrile nonhemolytic transfusion reaction
OR: odds ratio
pRBC: packed red blood cells
RBC: red blood cell
SFP: stored frozen plasma
TACO: transfusion‐associated circulatory overload
TAD: transfusion‐associated dyspnea
TR: transfusion reaction
TRACS: Transfusion Reaction Small Animal Consensus Statement
TRALI: transfusion‐related acute lung injury
WB: whole blood

## 1 Introduction

Blood transfusions can be lifesaving for critically ill cats and, with improved access to feline blood products, the frequency of transfusions is increasing [1]. However, transfusions are not benign, with a recognized risk of morbidity or mortality caused by transfusion reactions (TRs) [2]. The previously reported incidence of acute TRs with feline blood products varies widely, ranging between 8.7% and 23% of transfusion events [3, 4, 5, 6, 7, 8]. Interpreting these studies to determine the optimal care for individual cats is complicated by the different TR definitions used, the specific cat population studied, and study design. The Association of Veterinary Hematology and Transfusion Medicine (AVHTM) published a Transfusion Reaction Small Animal Consensus Statement (TRACS) with the aim of providing accurate definitions of individual clinical TRs and grading each with imputability criteria (definite, probable, or possible TR) [2, 9, 10]. Furthermore, consensus guidelines for preventing, monitoring, and treating TRs were presented, alongside identifying areas for further study [2, 9, 10]. Since the publication of TRACS, a survey of TRs in dogs using the new definitions has been published [11], but a comparable study in cats has not.

Our primary aim was to prospectively document the incidence and clinical characteristics of acute TRs, using TRACS definitions in cats receiving feline blood products [2]. Secondary aims were to assess whether specific transfusion factors, complications, and practices, including age and dose of red blood cell (RBC) products, donor unit blood type, cross‐matching, use of premedication, and method of administration were associated with TR incidence. Finally, we aimed to determine survival to 24 h after transfusion.

## 2 Materials and Methods

### 2.1 Cases and Data Collection

Fourteen referral hospitals, located in the United States of America (seven hospitals), United Kingdom (six), and Australia (one), prospectively logged all feline blood product transfusions administered over a 22‐month period (March 2022 to January 2024). Transfusions were identified through the collection of transfusion monitoring sheets, blood product storage data, charges on practice management systems, and manual checking of each individual unit's data. Each transfusion event was recorded using an online questionnaire (Data S1). Briefly, this questionnaire recorded recipient weight; any premedication (defined as a pharmaceutical agent administered before the transfusion with the explicit intention of decreasing the risk or severity of a TR); indication for transfusion; blood product collection date; and transfusion details including duration, volume of product administered, transfusion pauses, administration method, pre‐transfusion cross‐matching, whether the product was AB type‐matched, whether the cat was under general anesthesia (GA) at any point during the transfusion, and survival to 24 h post‐transfusion. Potential TRs were logged by recording the presence of any of the following (based on TRACS descriptions of an acute TR): acute fever > 39.2°C and a 1°C increase in temperature from the initiation of the transfusion; acute respiratory distress; acute tachycardia or hypotension (systolic blood pressure < 100 mmHg, with a decrease of at least 30 mmHg from baseline) or both; angioedema, urticaria, or pruritus; change in mentation; acute and new onset vomiting; acute and new onset diarrhea; or ionized hypocalcemia. Any other findings the clinician suspected to be consistent with a possible TR were recorded [2, 10]. For any potential reaction, follow‐up questions were asked following algorithms developed by TRACS, including evaluating for the presence of hemolysis, cardiovascular deterioration, or evidence of volume overload [10]. Treatments or changes to the transfusion plan after a TR were documented.

A transfusion event was defined as the administration of one blood product (a unit) from one donor to one recipient. Blood products were categorized as packed red blood cells (pRBC), whole blood (WB), or plasma products (subcategorized into fresh frozen plasma [FFP] and stored frozen plasma [SFP]).

Indications for using RBC‐containing products were grouped into the following categories: blood loss; hemolysis; ineffective erythropoiesis; extracorporeal treatment; volume or colloidal support or both; more than one indication; other; or unknown. Indications for the use of plasma products were grouped as follows: volume or colloidal support or both; coagulopathy; hemorrhage not caused by coagulopathy; extracorporeal treatments; more than one indication; other; or unknown.

### 2.2 TR Classification

Potential acute TRs recorded in the questionnaire were assessed by two authors (G.B.F.H., K.H.). Transfusion reactions were grouped into the following categories and subcategories: febrile nonhemolytic TR (FNHTR); respiratory reactions (transfusion‐associated dyspnea [TAD], transfusion‐associated circulatory overload [TACO], transfusion‐related acute lung injury [TRALI]); acute hemolytic TR (AHTR); allergic TR; citrate toxicity/hypocalcemia; and hypotensive TR. Each potential TR was examined and classified according to the case definitions and time frames set out by TRACS [2]. Imputability criteria (definite, probable, possible) were based on the individual TR definitions [2]. “Definite” cases fulfilled specific criteria for the individual TR, with no other cause for the clinical signs identified; “probable” cases either lacked specific data for a definitive classification or the transfusion was thought to be the most likely cause of the clinical signs, and “possible” cases were when other causes were considered more likely to be the reason for the findings but a TR could not be excluded. Follow‐up information was obtained as necessary. If two or more transfusions were given on the same day as a TR, the product received at the time of, or closest to, the TR was recorded as the unit associated with the TR. If two TR classes could explain the clinical signs seen in one transfusion, the records were analyzed and the TR with the highest imputability was used in the analysis. Only TRs with definite or probable imputability were included in the analysis to decrease the chance of miscategorizing clinical signs caused by another cause as a TR.

### 2.3 Statistical Analysis

Data analysis was performed using commercially available statistics software (SPSS version 29, IBM). Categorical data are presented as number (percentage), and continuous data as mean (SD) if normally distributed and median (25–75 percentiles) if not normally distributed. Where clinically pertinent, ranges were reported. For variables with missing data, the size of the denominator population was reported. Frequencies of TRs observed within the study sample are reported as percentages. Additionally, 95% Poisson confidence intervals (CIs) were calculated as estimates of population‐level TR incidence.

Associations between factors and TRs when giving RBC products were tested by logistic regression. Red blood cell (WB or pRBC) and plasma (FFP and SFP) products were grouped together to maximize statistical power. Associations were tested between blood product age (days of storage), dose (mL/kg), AB type matching, pre‐transfusion cross‐matching, prior transfusion status, method of administration, and a transfusion being given under GA, with any TR, FNHTR, AHTR, or allergic TR. Data were presented as odds ratios (95% CI). Factors that had *p* < 0.1 in univariable analysis then were included in a multiple logistic regression to assess for confounding factors, with subsequent adjusted odds ratios (aORs) presented. A sensitivity analysis was conducted removing the second of two TRs in any cat that had more than one TR, to assess if the inclusion of these TRs influenced measures of association between particular exposures and TR types. A further sensitivity analysis was conducted with site added to models to evaluate for confounding by institution. Fisher's exact tests were used to assess for significance in place of logistic regression whenever zero TRs occurred in one comparator group. *p* ≤ 0.05 were considered significant.

Ethical approval was granted for this study by the Royal Veterinary College University Teaching Hospital Clinical Research Ethical Review Board (URN‐2022‐2104‐03).

## 3 Results

### 3.1 Overview of the Study Group

A total of 608 transfusions were administered to 444 cats during the study period of March 1 2022 to January 31 2024. Of the 444 cats that received a transfusion, 117 received more than one transfusion. From 608 transfusion events, 41 TRs occurred in 39 cats, with two cats having two TRs associated with two separate transfusion events. Recipients were typed using one of two commercially available typing kits (Alvedia feline blood test quickTest and LabTest, France and RapidVet‐H feline blood typing, DMS laboratories, USA).

No TRALI, TAD, citrate toxicity/hypocalcemic, or hypotensive reactions were reported.

### 3.2 TR Incidence and Associations

#### 3.2.1 RBC‐Containing Products

A total of 513 RBC products (332 pRBC and 181 WB) were administered to 403 cats. More than one RBC product was received by 83 cats. The median time between RBC transfusions was 3 days (range, 0–346). Indications for RBC administration are listed in Table 1. Data regarding the characteristics of the RBC units and transfusion events are presented in Table 2. The median volume administered was 9.2 mL/kg (range, 0.5–41.7), and the median age of a unit was 9 days (range, 0–45). Units were administered via gravity flow in 1 case, infusion pumps in 24 cases, and syringe drivers in 483 cases. Three AB cats received type A blood (Table 2).

**TABLE 1:** Reported indications for 513 RBC product transfusions in 403 cats.

Indications for administration of RBC products	Number	%
Ineffective erythropoiesis	178	34.7
Hemolysis	137	26.7
Blood loss	123	24
More than one indication	37	7.2
Volume or colloidal support	2	0.4
Extracorporeal therapy	2	0.4
Unknown	34	6.6
	513	100

**TABLE 2:** Descriptive characteristics for transfusions of RBC‐containing products.

	Number/denominator	%
Received a RBC containing transfusion > 48 h before	121/472	25.6
AB type matched unit	507/510	99.4
Major cross‐match performed	272/485	56.1
Major cross‐match compatible	263/270	97.4
Syringe driver administration	483/508	95.1
Infusion pump administration	24/508	4.7
	*Median (IQR)*	
Volume administered (mL/kg)	9.2 (6.6–13.0)	
Age of unit (day)	9.0 (2–18)	

Abbreviations: IQR = interquartile range, RBC = red blood cell.

Transfusion reactions occurred in 40/513 RBC product transfusions, giving an incidence of 7.8% (95% CI, 5.6–10.6). The most common reactions were FNHTR (5.7%; 95% CI, 3.8–8.1), TACO (0.8%; 95% CI, 0.2–2.0), allergic (0.8%; 95% CI, 0.2–2.0), and AHTR (0.6%; 95% CI, 0.1–1.7).

Administering RBC‐containing products by infusion pump rather than a syringe driver was associated with an increased odds of FNHTR (aOR, 4.71; 95% CI, 1.43–15.54; *p* = 0.01). Increased age of unit (aOR, 1.04; 95% CI, 1.00–1.07; *p* = 0.05) also was associated with increased odds of FNHTR. These findings were not changed by sensitivity analyses. No other factors were associated with changed odds of a TR (Table 3).

**TABLE 3:** Association between potential transfusion reaction risk factors and the incidence of all, febrile nonhemolytic, acute hemolytic and allergic transfusion reactions associated with feline red blood cell‐containing transfusions in all study cats.

Outcome	Risk factor	Odds ratio comparison	# TRs/total in group	Univariable, OR (95% CI)	*p*	Multivariable, aOR (95% CI)	*p*
*Any TR*							
	Transfusion naivety	RBC ≥ 2 days previously vs. Not	8/121 vs. 29/351	0.79 (0.35–1.78)	0.56		
	Major cross‐match performed	Yes vs. no	21/272 vs. 15/213	0.91 (0.46–1.80)	0.78		
	Cross‐match compatible	Yes vs. no	19/263 vs. 2/7	0.20 (0.04–1.07)	0.06		
*n* = 40	Administration method	Pump vs. syringe driver	4/24 vs. 35/483	2.56 (0.83–7.90)	0.10		
	Volume administered	Per 1 mL/kg increase		1.02 (0.96–1.09)	0.45		
	Age of unit	Per day of storage		1.01 (0.98–1.05)	0.40		
*FNHTR*							
	Transfusion naivety	RBC ≥ 2 days previously vs. not	4/121 vs. 22/351	0.52 (0.17–1.52)	0.23		
	Major cross‐match performed	Yes vs. no	14/272 vs. 11/213	1.00 (0.45–2.26)	0.99		
*n* = 29	Cross‐match compatible	Yes vs. no	13/263 vs. 1/7	0.31 (0.04–2.79)	0.30		
	Administration method	Pump vs. syringe driver	4/24 vs. 24/483	3.83 (1.21–12.07)	0.02**	4.71 (1.43–15.54)	0.01**
	Volume administered	Per 1 mL/kg increase		0.99 (0.93–1.08)	0.98		
	Age of unit	Per day of storage		1.04 (1.00–1.07)	0.04**	1.04 (1.00–1.07)	0.05**
*AHTR*							
	Transfusion naivety	RBC ≥ 2 days previously vs. not	0/121 vs. 3/351	n/c			
	Major cross‐match performed	Yes vs. no	1/272 vs. 2/213	2.57 (0.23–28.52)	0.44		
*n* = 3	Cross‐match compatible	Yes vs. no	0/263 vs. 1/7	n/c			
	Administration method	Pump vs. syringe driver	0/24 vs. 3/483	n/c			
	Volume administered	Per 1 mL/kg increase		1.00 (0.79–1.26)	0.97		
	Age of unit	Per day of storage		n/c			

*Note:* Odds ratios, adjusted odds ratios, and *p* values from single variable and multiple logistic regression.

Abbreviations: # = number, AHTR = acute hemolytic transfusion reaction, aOR = adjusted odds ratio, FNHTR = febrile nonhemolytic transfusion reaction, n/c = non‐calculable, OR = odds ratio, RBC = red blood cells, TR = transfusion reaction.

** Statistically significant associations (*p* ≤ 0.05).

Cross‐match data were available for 485 transfusion events, with 272 cats having a major cross‐match performed. Seven known cross‐match incompatible units were administered, with a single AHTR and FNHTR occurring in two recipients. Data were available for 119 cats that had received a second transfusion 2 or more days after a previous RBC transfusion regarding cross‐match information. Of these, 106 had a cross‐match performed and 13 did not. No TR occurred in these 13 cats. For transfusion‐naïve cats, data on cross‐matching were available for 346 transfusions, with 160 having a cross‐match performed. Prior transfusion status was not known in six cats. Performing a cross‐match in a transfusion‐naïve cat was not associated with a decreased odds of any TR (OR, 0.99; 95% CI, 0.46–2.15; *p* = 0.98), FNHTR (OR, 0.94; 95% CI, 0.39–2.28; *p* = 0.90), or AHTR (OR, 1.73; 95% CI, 0.16–19.2; *p* = 0.66). Six of the 160 transfusion‐naïve cats received units with a known incompatible major cross‐match. Of these, one FNHTR and one AHTR were documented. Three AB cats received a RBC transfusion from a type A donor, and a single TR (TACO) was documented.

#### 3.2.2 Plasma Products

Ninety‐five plasma units were administered to 69 cats. Fresh‐frozen plasma was the most frequently administered plasma product (74), followed by SFP (21). One cat with AB blood type received type A plasma twice, with no reaction. Twenty‐one cats received > 1 unit of a plasma product. Indications for plasma transfusions are shown in Table 4. The median volume administered was 7.1 mL/kg (range, 2.9–21.1). A single FNHTR occurred, giving an incidence of 1.1% (95% CI, 0.0–5.9). No other TRs were recorded.

**TABLE 4:** Reported indications for 95 plasma product transfusions in 69 cats.

	Number	%
Volume or colloidal support	64	67.4
Hemorrhage	11	11.6
Coagulopathy	13	13.7
More than one indication	4	4.2
Extracorporeal therapy	3	3.2
Total	95	100

### 3.3 TRs—Clinical Presentations and Management

#### 3.3.1 FNHTRs

There were 30 FNHTRs, 29 with RBC‐containing product and one with plasma. The median maximum body temperature was 39.6°C (range, 39.2°C–41.1°C). Transfusions were paused to evaluate for a TR in 12 cases, with three not being restarted. One cat received an antihistamine and one had the transfusion stopped; the remainder received either no treatment, had the rate of the transfusion slowed, or both.

#### 3.3.2 Allergic TR

Of the four cases of allergic TRs, three had acute and new‐onset gastrointestinal signs (two vomiting and one diarrhea), and one developed acute facial angioedema. Respiratory type I hypersensitivity reactions were not documented. Two transfusions were paused for a TR evaluation. Maropitant was given to cats with gastrointestinal signs, and an antihistamine was administered to the cat with cutaneous signs. All cats completed their transfusions.

#### 3.3.3 Acute Hemolytic TRs

Of the three cats that displayed AHTRs, two were febrile (≥ 39.2°C) with an increase in body temperature of at least 1°C during the transfusion. Neither of these cats had a cross‐match performed before transfusion, and neither of them had their transfusions stopped. The third cat was knowingly given an incompatible major cross‐matched unit (as all available units were cross‐match incompatible) and experienced cardiovascular deterioration with an associated decrease in PCV. The affected cat did not develop pyrexia. The transfusion was stopped and not restarted. One febrile cat received an antihistamine. All cats were transfusion‐naïve and received AB type‐specific blood.

#### 3.3.4 Respiratory Reactions

Four TACO reactions were documented, two with pRBC and two with WB. No cats had their transfusion paused. Product dose ranged from 8.3 to 19.3 mL/kg. Furosemide was administered in two cases. No cases of TAD or TRALI were recorded.

### 3.4 Transfusion Practices and Other Complications

#### 3.4.1 Blood Product Time at Room Temperature, Pauses During Administration and Transfusion Duration

Blood products were at room temperature for > 4 h in 9% of the transfusion events. The median time to administer the whole transfusion was 4:00 h (range, 3:03–5:54), including units that were divided and stored appropriately to enable a longer transfusion duration.

Data regarding pauses were available in 608 cases with 63 (10.4%) transfusions paused. Forty‐three (68.3%) of these were restarted with completion of the transfusion. The most frequent reason for pausing a transfusion was to assess for a potential TR (28/608, 4.6%), followed by an administration complication, such as cannula loss or pump malfunction (29/608, 3.1%), performing diagnostic tests (6/608, 1.0%), administering other treatment (4/608, 0.7%), and death (3/608, 0.5%). Of the 28 pauses associated with TR evaluation, 13 (46.4%) did not fulfill any TRACS criteria for a TR. Six cats with suspected TRs had their transfusions stopped, and 50% of these did not match any TRACS criteria for a TR.

#### 3.4.2 Premedication

Premedication was administered with the aim of decreasing the likelihood of a TR in two cats. One received an antihistamine and one maropitant. No further analysis was performed because of the low frequency of premedication.

#### 3.4.3 General Anesthesia

Forty‐five cats received a transfusion while under GA for all or part of the transfusion. No TRs were detected in these recipients.

#### 3.4.4 Death Within 24 h of Transfusion

Data regarding 24‐h survival was available for 596 in transfusion events in 436 cats. Eighty cats (18.3%) died within 24 h of receiving 96 transfusions. Of the non‐survivors, three cats had three transfusions in the prior 24 h, seven cats two transfusions, and 70 a single transfusion. Documentation of a TR was not associated with the odds of mortality in the following 24 h (OR, 0.76; 95% CI, 0.29–1.99; *p* = 0.58).

## 4 Discussion

The overall TR incidence in cats in this cohort, as defined by recently published consensus definitions [2], was 7.8% for RBC‐containing products and 1.1% for plasma products.

Febrile nonhemolytic reactions predominated, with an incidence of 5.7% (95% CI, 3.8–8.1) for RBC‐containing products, consistent with the lower end of previous incidence reports, which have ranged from 4% to 22.9% [5, 6, 7, 8, 12]. The requirement for an FNHTR to increase by 1°C and to be > 39.2°C is a more stringent definition than those used in studies that report higher incidences [5, 8]. A single FNHTR was the only TR documented during the 95 plasma transfusion events, giving an incidence for both all TRs and FNHTR of 1.1% (95% CI, 0.0–5.9) for this product type. Previous reports of TR incidence in cats receiving plasma are much higher than in our study, with ranges from 14.8% to 16% for all acute TRs and 3.7% to 10% for FNHTRs [3, 4]. The lower rate in our study again is likely a consequence of the more stringent and precise criteria used.

The 0.8% incidence of TACO is comparable with the lower end of ranges reported for volume overload or TACO in cats, reported to be 0.7%–3% [5, 6, 7]. The variation in incidence is likely a result of differences in definitions among studies. One study included cats with TACO that experienced documented respiratory distress within 24 h of a transfusion [6], in comparison to the 6 h in TRACS [2]. Furthermore, in that study, the clinical signs of TACO were defined as an increase in respiratory effort and tachypnea, rather than the more stringent TRACS criteria including worsening respiratory signs, dyspnea, orthopnea, pulmonary crackles, or cough [2, 6]. Severely anemic cats have an increased risk of left heart volume overload, and in one study evidence of overload was detected by echocardiography within 24 h after transfusion [13, 14]. It therefore is unclear whether the 6 h window in the TRACS definition is too short a time frame for cats to demonstrate clinical signs consistent with volume overload.

Previous reported incidences of AHTRs in cats range between 0.4% and 6.9% [6, 7, 8, 12]. The incidence of 0.6% in our study is comparable with a large retrospective study [12], and lower than that reported in prospective studies assessing cross‐match compatibility [6, 7, 8]. Our study might have underestimated AHTRs compared with prospective studies that were actively monitoring the study population for hemolysis. Although the TRACS algorithm recommends investigation for hemolysis (e.g., using a centrifuged microhematocrit tube to observe plasma color) after the development of a fever or cardiovascular or respiratory signs, and these evaluations were not always performed [2]. This omission could have led to AHTRs being missed or erroneously classified as FNTHRs. Our study emphasizes the need for increased awareness and appropriate monitoring and action when a suspected TR occurs to better detect AHTRs and optimally treat cats.

The incidence of allergic reactions in cats in transfusion studies varies between 0% and 3.7%, with several studies not reporting such adverse events [3, 5, 6, 7, 8, 12, 15]. Studies with higher incidences have used broader criteria, such as including inappetence [3, 7]. The incidence of 0.8% documented here, using more precise definitions, suggests that cats experience a lower frequency of allergic TRs as compared with dogs [11, 16, 17].

Secondary aims of our study were to evaluate transfusion factors associated with TRs, document current transfusion practices, and assess survival at 24 h after transfusion. The odds of an FNHTR were higher with increasing RBC‐containing unit age (aOR, 1.04 per day). This result is similar to previous studies in dogs and cats that have shown a positive association with increased odds of any TR, FNHTRs, and AHTRs with increased age of RBC units [5, 11, 18].

Administering the transfusion by infusion pump rather than syringe driver was associated with increased odds of FNHTR (aOR, 4.71). The etiopathogenesis for an FNHTR occurring secondary to infusion pump use is not clear. Possible explanations could include pyrogens being stimulated by larger numbers of RBC microparticles formed as fragile feline RBCs pass through an infusion pump, or other undetermined mechanisms. Alternatively, it may be that some cats developed pyrexia before clinically detectable hemolysis, resulting in misclassification of AHTRs as FNHTRs. The destruction of red cells by non‐immunological mechanisms, such as mechanical forces transmitted by an infusion pump, would lead to an AHTR. Furthermore, the infusion pump mechanism (piston or peristaltic) may alter the extent of hemolysis generated, although a recent ex vivo study failed to document a difference in hemolysis rates between feline pRBC units administered by gravity, syringe pump, or two linear peristaltic infusion pumps [19, 20]. This difference in TR with administration methods was not found in a large multicenter prospective study of transfusions in dogs, which could be a result of species differences in RBC characteristics [11]. It is also possible that the finding in cats represents type I statistical error. Regardless of the cause, this finding supports the TRACS suggestion of using a syringe pump to administer RBC to cats [9, 19].

No TRs were detected under anesthesia. Given the predominance of FNHTRs in our study, this finding is not surprising. Any febrile response may be masked by hypothermia that may occur during GA. Indeed, no FNHTRs were recorded in dogs under GA in recent prospective and retrospective transfusion studies [11, 21].

The most common indications for administering blood products, most notably plasma, are different from those previously reported [3, 4, 5, 6]. Plasma was administered for volume or colloidal support or both in 67.4% of cases, for the treatment of underlying coagulopathy in 13.7%, and for blood loss in 11.6%. Recent retrospective studies of plasma use in cats reported coagulopathies as an indication in 83%–95% of cases, with hypotension being the indication in 25% of cats in one study and hypoalbuminemia in 5% in the other [3, 4]. Our study therefore emphasizes a shift in prevailing attitudes regarding the use of plasma products in transfusion medicine in cats, with a shift towards using plasma as a natural colloid and for volume support. This shift in practice may be associated with increased awareness of the potential risks of using synthetic colloids in cats and the lack of feline albumin products [22]. Ineffective erythropoiesis was the most common reason to administer RBC products in our study. A previous study documented a similar finding in a study of 450 transfusion events in cats [5]. However, other studies have found that blood loss or hemolysis are the leading indications for transfusion of RBCs [6, 7]. These differences might reflect variation in case load among hospitals or a progressive change in transfusion practices [5, 6, 7].

Only 9% of blood products administered were at room temperature for > 4 h, an improvement over the 19% documented in a prospective study of transfusions in dogs from the same centers [11]. This change likely reflects the increased practice of administering transfusions to cats using syringe drivers rather than infusion pumps or gravity, enabling part of a transfusion to remain refrigerated if a pause is required or if the recipient would benefit from a slower administration rate, and avoiding delays associated with positional changes impairing gravity flow. Of the 10.3% transfusions that were paused, 44.4% of these were for evaluation after suspicion of a TR, and 46.4% of these did not fulfill TRACS criteria for a TR. Although prudence is warranted during the administration of blood products, it is concerning that 50% of the transfusions that were discontinued because of possible TRs failed to meet TRACS classification for a TR. Our study therefore emphasizes the importance of increasing knowledge about TR detection, monitoring, and treatment to avoid wasting feline blood products.

The mortality rate within 24 h of a transfusion event was relatively high at 18.3%, likely reflecting the critical condition of many of the cats receiving blood products. Non‐survival to discharge was most recently reported at 25.5% in cats receiving transfusions, and although not directly comparable, this population is likely similar to that reported in our study [5].

Our large prospective multicenter study using evidence‐based consensus‐derived definitions had many strengths. The use of TRACS imputability statements, two authors classifying the TRs, and the exclusion of “possible” TRs enhance the accuracy of the incidences reported. However, multiple authors logged the data in the survey, and despite supportive information being sought, such information was not always available, meaning that TRs could have been misclassified, given a lower imputability grade, or missed. The incidence of TRs was low, decreasing the ability of the study to detect small differences among groups. Most notably, cross‐matching information was not always available. Similarly, the brands of infusion pumps, if used, were not recorded. Finally, the use of medication, such as glucocorticoids in some cats with immune‐mediated disease, might have altered the clinical signs manifested by the cats and decreased the detection of a TR.

## 5 Conclusion

Transfusion reaction incidence in cats, as defined by TRACS consensus‐derived and evidence‐based guidelines, as described in our study can serve as a benchmark for future transfusion studies in cats. Febrile nonhemolytic transfusion reactions were the most common TR in cats, with an incidence of 5.7% in RBC‐containing products and 1.1% in plasma products. An increased odds of FNHTR was associated with increasing RBC unit age and administering RBCs by infusion pump, in comparison with a syringe driver. These observational findings provide evidence for clinicians to optimize decision making for cats under their care and modify the risk of a TR. Increasing awareness of TR definition, risks, and management will decrease unnecessary wastage of feline blood products and increase the ability of clinicians to communicate the benefits and risks of transfusions to clients.

## Disclosure

Authors declare no off‐label use of antimicrobials.

## Ethics Statement

Approved by Royal Veterinary College University Teaching Hospital Clinical Research Ethical Review Board (URN‐2022‐2104‐03). Authors declare human ethics approval was not needed.

## Conflicts of Interest

The authors declare no conflicts of interest.

## Supporting information


**Data S1:** Transfusion monitoring.
