# Supplementary material for: A Prospective Multicenter Observational Study Assessing Incidence and Risk Factors for Acute Transfusion Reactions in Cats
Source: J Vet Intern Med. 2025 Sep 29;39(6):e70246. doi: 10.1111/jvim.70246 (PMC12477435; doi:10.1111/jvim.70246)
Supplement: Supplementary file 1 — Data S1: Transfusion monitoring. [file JVIM-39-e70246-s001.pdf]

Online surveys

Transfusion Monitoring

Showing 0 of 0 responses

Showing **all** responses

Showing **all** questions

1 What is the name of the institution where this transfusion was administered?

RVC	0
RDVS	0
Willows	0
MS State	0
Auburn	0
Tufts	0
Langford	0
DWR	0
Queensland	0
Georgia	0

2 What species was the recipient?

Option	Count
Dog	0
Cat	0

3 What is the unique case identifier (case number) of the recipient?

No responses

4 What blood product was adminstered?

Option	Count
PRBC	0
FFP	0
Whole blood	0
Non-FFP Whole plasma	0
Other	0

4.a If you selected Other, please specify:

No responses

5 What was the date of blood product collection?

No responses

6 Was the blood product leukoreduced prior to storage?

Option	Count
Yes	0
No	0
Unsure	0

7 Was a drug administered prior to the transfusion to decrease the risk of trasnfusion reaction (pre-treatment)?

Option	Count
Yes	0
No	0

7.a What drug was administered?

Option	Count
Acetaminophen	0
Anti-histamine	0
Corticosteroid	0
Other	0

7.a.i What drug was administered?

No responses

8 Please provide the date and time of the start of the blood product transfusion

No responses

9 Which of the following methods of adminstration was used?

Option	Count
Fluid pump	0
Gravity	0
Syringe driver	0
Other	0

9.a Please specify method of administration

No responses

10 Were any of the following noted during the period of transfusion administration or in the 24 hours after the end of the transfusion? Tick all that apply.

Option	Count
Acute fever over 39C (102.5F) AND 1C (1.8F) above temperature at transfusion starting point	0
Acute respiratory distress	0
Acute tachycardia and/or hypotension	0
Angioedema/urticaria/pruritis	0
Change in mentation	0
Hypocalcaemia	0
Hypothermia	0
Vomiting (acute and new onset)	0
Diarrhoea (acute and new onset)	0
Other possible transfusion reaction	0
None of the above	0

10.a Was plasma haemolysed at the time of tachycardia/hypotension?

Option	Count
Yes	0
No	0
Not checked	0

10.a.i Was the patient

Option	Count
Receiving a massive transfusion? (1 whole blood volume in 24hours, 90ml/kg dogs 60ml/kg cat)	0
Having on-going severe haemorrhage?	0
Showing signs of anaphylaxis? (Tachycardia, hypotension, GI signs and/or gall bladder wall oedema)	0
Showing acute respiratory distress?	0

10.a.ii Please give further information about: a. The severity of tachycardia/hypotension b. When the tachycardia/hypotension was noted c. Any further investigation performed not detailed previously d. Any treatment administered

No responses

10.b Were new bilateral pulmonary infiltrates present on CT, radiography or ultrasound?

Option	Count
Yes	0
No	0
Unsure	0
Not checked for	0

10.b.i Was there evidence of plasma haemolysis at the time of respiratory distress?

Option	Count
Yes	0
No	0
Not checked for	0

10.b.ii Was there evidence of cardiac overload on echocardiography or NT-proBNP measurement?

Option	Count
Yes	0
No	0
Not checked	0
Other	0

0.b.ii.a If you selected Other, please specify:

No responses

0.b.ii.b Please give details of echo measurements or NT-proBNP value if taken.

No responses

.0.b.ii.c Please give further information about: a. The respiratory distress b. When the respiratory distress was noted c. Any further investigation performed not detailed previously d. Any treatment administered

No responses

10.c Was there plasma haemolysis at the time of the fever?

Option	Count
Yes	0
No	0
Not checked	0
Unsure	0

10.c.i Was acute respiratory distress noted?

Option	Count
Yes	0
No	0

10.c.i.a Did the patient have acute cardiovascular deterioration at the time of the fever?

Option	Count
Yes	0
No	0

).c.i.a.i Please give details of severity and parameters

No responses

.c.i.a.ii Please give further information about: a. The peak fever b. When the fever was noted c. Any further investigation performed not detailed previously d. Any treatment administered

No responses

10.c.ii Was there evidence of

Option	Count
Incorrect blood type	0
Cross match incompatibility	0
Out of date unit	0
Inappropriate transfusion administration set up	0
Other concern with unit or administration method	0
None of the above	0

.0.c.ii.a Please give further details about suspected incompatability or inappropriate administration method.

No responses

.c.ii.a.i Please give further information about: a. The peak fever b. When the fever was noted c. Any further investigation performed not detailed previously d. Any treatment administered

No responses

10.d Please give further information about: a. The clinical signs seen b. When the clinical signs were seen c. Any further investigation performed d. Any treatment administered.

No responses

11 Was the transfusion paused at any time?

Option	Count
Yes	0
No	0

11.a Please give details about why the transfusion was paused and how long the transfusion was stopped for.

No responses

12 Was the whole planned transfusion volume administered?

Option	Count
Yes	0
No	0

12.a Please give details as to why planned volume was not administered.

No responses

13 Please provide the date and time of the end of the transfusion

No responses

14 Did any of the following occur during the transfusion?

Option	Count
Blood product at room temperature for greater than 4 hours	0
Intravenous catheter displacement	0
Incorrect blood product rate administration	0
Other transfusion associated complication	0
None of the above	0

14.a Please give more details about the complication including its timing and any actions taken.

No responses

15 What was the underlying disease process meaning blood product transfusion was required?

Option	Count
Anticoagulant rodenticide induced coagulopathy	0
Bone marrow disease	0
IMHA / PIMA	0
IMTP	0
Trauma induced blood loss	0
Other	0
Unknown	0

15.a Please give more details about the underlying disease process

No responses

16 Was the patient alive at 24 hours post transfusion

Option	Count
Yes	0
No	0

17 Did the patient have another blood product transfusion in the 24 hours prior to or 24 hours after this blood product transfusion

Option	Count
Yes	0
No	0

17.a Have you registered the other transfusion(s) or are you planning to do so immediately after this entry?

Option	Count
Yes	0
No	0
